# Rift Valley Fever Encephalitis

**DOI:** 10.3201/eid1003.020817

**Published:** 2004-03

**Authors:** Abdulrahman A. Alrajhi, Abdulaziz Al-Semari, Jehad Al-Watban

**Affiliations:** * King Faisal Specialist Hospital and Research Centre, Riyadh, Saudi Arabia

**Keywords:** Rift Valley fever Virus, encephalitis, retinitis, Saudi Arabia

**To the Editor:** Rift Valley fever (RVF) is an undifferentiated febrile illness caused by Rift Valley fever virus (RVFV). Several human outbreaks occurred in Africa, resulting in tens of thousands of infections (*1*,*2*). During fall 2000, an outbreak of RVF in the Arabian Peninsula (the first recorded outside of Africa) resulted in many human and animal fatalities (*3*,*4*). Severe and frequently fatal encephalitis thought to be directly related to viral invasion of the central nervous system develops in <1% of patients (*5*). Encephalitis complicating RVF is poorly described in the literature, which offers no detailed description of the clinical findings, results of cerebrospinal fluid (CSF) studies, or imaging. We describe a case of RVF encephalitis associated with retinitis, including CSF findings, viral culture results, and neuroradiology findings

An 18-year-old woman from Jazan (southwest of Saudi Arabia) had a 3-day history of confusion, fever, and blurred vision at the time an RVF outbreak was peaking in Jazan. Philadelphia-chromosome–positive chronic myeloid leukemia (CML) had been diagnosed in her several months before (leukocyte count 108 x 10^9^/L). She responded well to hydroxyurea, and she had been in stable-phase CML for a few months. At the time of her visit, her temperature was 39.2°C, blood pressure 110/70 mm Hg, pulse 120 beats/min, respiratory rate 22/min. She had no lymphadenopathy, pallor, or jaundice. Results of her head, neck, and throat examinations were normal. Her chest was clear, and her abdomen was soft and nontender. Her spleen was 3 cm below the left costal margin. She was conscious and oriented. No meningeal signs could be elicited. Pupils were equally reactive to light with normal extraocular movements. Extremities had normal tone, power, sensation, and reflexes. Plantar reflex was flexor bilaterally. She had ataxic gait and bilateral retinal hemorrhages. She was unable to count fingers. Hemoglobin was 100 g/L, leukocyte count 5.1 x 10^9^/L, and platelets 373 x 10^9^/L. Renal and liver function tests were normal. Contrast-enhanced computed tomography (CT) scan of the brain was normal. Urine analysis and malaria smear were negative. CSF was clear. CSF glucose was 3.9 mmol/L (serum 5.8), protein 455 mg/L. CSF leukocyte count was 323 x 10^6^/L, 58% lymphocytes, and 38% polynuclearleukocytes. Tests for hepatitis B surface antigen, antibodies to hepatitis C virus, HIV, cytomegalovirus antigenemia, rheumatoid factor, and antinuclear antibodies were negative. Cultures from blood, CSF, and urine were negative for bacteria. CSF viral culture was negative. Polymerase chain reaction for herpes simplex virus and enterovirus from CSF was negative. Tests for serum anti-RVF virus immunoglobulin M were positive. No other tests for RVFV were performed. Bone marrow on admission day was consistent with CML in remission. Prednisone was started on admission for 7 days.

On hospital day 5, the patient was noted to be agitated, confused, and unresponsive to commands. She was transferred to the intensive care unit after her level of consciousness decreased; she was moving all four limbs but did not respond to verbal commands or painful stimuli. Pupils were 5 mm equal bilaterally with a sluggish reaction to light. Corneal reflexes were reactive bilaterally. Gag reflex was present, and tone was increased in all four limbs with brisk reflexes and extensor planter responses. The next day, her condition deteriorated, and she became unresponsive to painful stimuli. Repeated CT scan of the brain showed no pathologic changes. Electroencephalogram showed generalized continuous rhythmic sharp and spike wave activity consistent with nonconvulsive status epilepticus. Magnetic resonance imaging (MRI) of the brain showed bilateral frontoparietal high signal intensity on T2-weighted images and evidence of subtle right posterior thalamic hyperintensity with no corresponding abnormalities in T1-weighted images. The axial diffusion MRI images were more elaborative, showing multiple bilateral asymmetrical cortical hyperintense areas consistent with an ischemic or inflammatory process. Phenytoin was started. The next day, the patient was able to open her eyes and responded to painful stimuli, corneal reflexes were present bilaterally, oculocephalic reflex was present, and planters were flexors bilaterally. Repeat CSF studies on day 30 showed glucose of 3.8 mmol/L, protein 431 mg/L, leukocyte count of 12 x 10^6^/L, and 68% polynuclearleukocytes. Repeat CT scan of the brain showed new bilateral temporo-occipital hypodensity more on the left side and a probable right middle cerebellar and left thalamic internal capsule infarct. There was no intracranial hemorrhage or hydrocephalus. She was discharged home awake, blind, quadreparetic, and incontinent, on anticonvulsants. After 1 year, her neurologic condition had not changed.

Encephalitis and retinitis are severe complications of RVF, developing 1 or 2 weeks into the course of diseases. By that time, RVFV antigen assay is negative. Our patient met the definition of an RVF case during the outbreak (*3*). In one outbreak in Mauritania, 4.9% of observed infections had encephalitis (*6*), although the true frequency of encephalitis in RVF may be overestimated because infection can go unrecognized. The literature contains limited clinical description of this syndrome (*6*–*8*). Detailed neuroimaging findings, including MRI and flow studies, have not been previously reported. These findings, along with the patient’s clinical signs and symptoms, suggest cerebral vasculitis; however, no angiogram was performed, and markers of vasculitis were negative. A more likely cause would be direct viral parenchymal invasion.

The pathogenesis of RVF encephalitis in humans is not clear. Animal studies indicate that active viral replication and necrotizing encephalitis with diffuse perivascular infiltrates of lymphocytes and macrophages occur in cerebral parenchyma (*9*,*10*). Postmortem histopathologic examination of brains of fatally infected rhesus monkeys have shown a mild, nonsuppurative, multifocal, perivascular encephalitis in the cerebral cortex, primarily of lymphoplasmacytic cells and nodular aggregates of neutrophils in association with mild necrotic changes of neurons (*11*). In one patient who died of meningoencephalitis in South Africa, brain pathologic findings showed perivascular cuffing and round-cell infiltration (*2*). In humans, we are not aware of positive RVFV cultures from CSF, blood, or brain during encephalitis. We have no reason to suspect CML to influence disease manifestations in this patient. CML was in stable phase for several months and should not have affected immune response of the patient towards RVFV.
